# Understanding Retinal Changes after Stroke

**DOI:** 10.4236/ojoph.2017.74037

**Published:** 2017-11-06

**Authors:** Varun Kumar

**Affiliations:** Department of Ophthalmology, School of Medicine, Stanford University, Stanford, CA, USA

**Keywords:** Stroke, Retinal-Ischemia, Animal Models

## Abstract

Stroke is the fifth leading cause of death and disability in the United States. According to World Heart Federation, every year, 15 million people suffer from stroke worldwide out of which nearly 6 million people die and another 5 million people are disabled. Out of many organs affected after stroke, one of them is eye. Majority of the stroke victims suffer vision loss due to stroke-induced retinal damage. However, stroke-induced retinal damage and microvascular changes have not been given paramount importance in understanding stroke pathophysiology and predicting its occurrence. Retinal imaging can be a very powerful tool to understand and predict stroke. This review will highlight the importance of retinal changes in predicting occurrence of stroke, major retinal changes, the relationship between retinal diseases and stroke and moreover, molecular mechanisms delineating the stroke induced-retinal changes and therapeutics associated with it.

## 1. Introduction

Stroke is one of the leading causes of adult death and disabilities in the world. According to American Heart association, it is the fifth leading cause of death and long term disability in the United States [1]. Approximately 800,000 people in the USA suffer from stroke every year. 87% percent of stroke is ischemic whereas, 13% is hemorrhagic. Only FDA approved treatment for stroke is tissue plasminogen activator (tPA), which is a thrombolytic agent. However, it can be used effectively only within 4 – 5 hours after stroke which is not always feasible. Therefore, better therapeutic targets should be investigated. Moreover, detection of early pathological changes after stroke can lead to new therapeutic targets. However, early detection becomes challenging due to difficult access, imaging and complex pathophysiology of the affected area after stroke. Therefore, there is an unmet need to investigate an area of brain, which is similar in vasculature and physiological changes, easily accessible and can be examined with noninvasive or minimally invasive methods so that such organ may become a direct window to the cerebral pathology. Eye, being one such part of brain, projects out of diencephalon, has similar embryonic origin, vasculature, blood barriers and pathophysiology. Various studies have suggested that there are specific changes associated with neurological diseases detected at a very stage of the disease in the retina of eye. For example, there is an aggregation of toxic huntingtin protein [2], intra retinal amyloid deposition [3], loss of retinal dopaminergic neurons [4] in mouse model of Huntington, Alzheimer and Parkinson’s disease respectively. Specifically, stroke is a cerebrovascular accident involving blood vessels. Therefore, understanding and imaging blood vessels is of paramount importance in understanding stroke. Retinal vessels can be easily accessed and examined using recent advanced imaging instruments such as optical coherence tomography angiography (OCTA), optical coherence tomography (OCT) etc. Moreover, stroke patients indeed demonstrate microvascular changes in retina. For example, ischemic stroke patients have sparser and tortuous microvascular network in the retina [5]. Therefore, retinal imaging can be a powerful imaging tool to detect and understand the early pathological changes and can even predict the occurrence of subsequent stroke. This review has been developed in a certain fashion as depicted in the flow chart diagram (Table 1) and summarizes important findings in the field of eye and stroke research (Table 2).

## 2. Retina for Investigating Physiological Changes after Stroke

As described earlier, retina being a part of brain, shares common embryonic origin, similar anatomy, vasculature, blood barrier and physiology thus, makes it a perfect fit for investigating earlier pathological changes after stroke. In this part, we are further extending the discussion about retina for investigating early pathophysiological changes after stroke. Ischemic injury is a complicated event caused by series of events such as inflammation, excitotoxicity, energy depletion etc. Such molecular events in the brain affects retina [6]. The similar pathophysiological changes in the retina also occur at molecular level in different parts of the brain [7]. Thus, eye and brain are interlinked and can certainly provide useful information for each other. But the question remains why can’t we image and understand the changes in the brain directly instead of imaging the eye? As described above, part of the reason is the availability of very sophisticated, time consuming brain imaging techniques such as MRI, magnetoencephalography etc. which makes it difficult to image brain directly. There is no doubt that they have contributed significantly in understanding stroke and its clinical symptoms. However, there has always been an issue with accessibility for imaging organs after stroke which might be used to predict early pathophysiological changes. This has not been investigated in more details. Retina is easily accessible and early physiological changes occurring in the brain after stroke is observed in the retina in a similar fashion. Thus, it can provide useful information about changes after brain stroke [8]. Moreover, retinal changes are observed in many diseases such as diabetic retinopathy, retinal arteriolar emboli, age related macular degeneration. All of these diseases increase the chances of ischemic stroke [8]. Moreover, as mentioned earlier, the retina extends out of the diencephalon and possesses a blood retinal barrier similar to blood-brain barrier [9]. Cerebral vessels after stroke have similar changes as observed in the retinal vessel changes and can predict occurrence of stroke [10]. For example, De Silva *et al*. demonstrated that patients with severe focal retinal arteriolar narrowing were highly susceptible to recurrent cerebrovascular events as compared to those without arteriolar narrowing [11]. Most importantly as described earlier, retinal vasculature can be non-invasively visualized easily *in vivo* after stroke. Moreover, retinal examination can also be useful for stroke risk stratification as well. For example, McGeechan *et al*., demonstrated that wider retinal venular caliber increases the risk of stroke in humans whereas the caliber of retinal arterioles was not associated with stroke [12]. They further put emphasis on considering inclusion of retinal venular caliber in prediction models containing stroke risk factors, which can reassign intermediate risk stroke category to lower risk. All the above studies strongly suggest that it will be worth understanding and imaging retina for early detection of pathophysiological changes after stroke. Moreover, this will be important step forward in improving treatments for stroke since, it can provide sufficient molecular information which can be targeted for therapeutics after stroke. Moreover, retinal damage after stroke has not been given paramount importance in the stroke treatment regime. However, the retinal damage caused by stroke and other complicated diseases is difficult to differentiate at this stage.

## 3. Relationship between Retinopathy Diseases and Stroke

Many diseases such as diabetic retinopathy, ischemic optic neuropathy, age related macular degeneration, hypertension etc. are often associated with the occurrence of stroke [13]. However, there are some conflicting studies as well. We will briefly discuss some of them. Diabetic retinopathy is one of the leading causes of blindness worldwide. Diabetes is a common risk factor for developing ischemic optic neuropathy [14]. However, there are some conflicting reports [15]. Moderate hypertensive retinopathy includes microaneurysm, cotton-wool spots, retinal hemorrhages and hard exudates. These have been associated with increased incidence of stroke [16] [17]. Even the mild hypertensive retinopathy signs such as focal retinal arteriolar narrowing, increased arteriolar reflux etc. have been implicated for increased risk for stroke. Retinal arteriolar emboli are plaque-like lesions in the lumen of the retinal arterioles composed of cholesterol fragments, fibrin aggregates or calcified valves. Some studies suggest that it is not associated with increased incidence of stroke but related with higher risk of stroke related death [18] [19]. Retinal vein occlusion indicates the presence of retinal hemorrhages, cotton wool spots, macular and optic disc edema. The disease increases with age but is not often associated with increased stroke incidence [20]. Age related macular degeneration is another chronic retinal disease and considered one of the leading causes of blindness worldwide. It affects the central area of the retina called macula. The main characteristics are soft drusen yellow deposits under retinal pigmental epithelium. The prevalence of this disease is associated with increased age. Several studies suggest the association between age related macular degeneration and stroke [21] [22] [23]. All these studies suggest that various ocular diseases associated with retinopathy increases the incidence of stroke [24]. However, the strength to which it contributes to stroke incidence remains inconclusive. The association between retinal signs and stroke has been evaluated in all stroke affected populations. However, there are no studies suggesting the specific stroke subtypes (ischemic or hemorrhagic) and retinal signs. In the future, various studies will further confirm the association between stroke and retinopathy. However, researchers need to develop a standardized photographic and grading classification to assess retinal diseases so that there is better comparison and confirmation of the findings. Moreover, as discussed above, there is strong association between retinopathy and stroke. Therefore, retinal photography can act as a surrogate marker of treatment efficacy, which might be quite useful in clinics.

## 4. Retinal Imaging Technology

Over the years, the use of light has been the key in revealing the structural and functional information from the human and rodent’s retina. New scanning and imaging technologies have contributed significantly in imaging and understanding the retinal blood vessels, nerve fiber layer and optic disc etc. These eye parts are mostly affected in many ocular as well as cerebrovascular diseases. Fundus photography has been the basis of imaging optic nerve and retina [25]. However, the lack of fine details generated in the fundus photography led to the additional ocular techniques such as stereo disc photography, red free retinal nerve fiber layer photography etc. There has been tremendous progress in the development of series of imaging ocular microscopes such as Confocal Scanning Laser Ophthalmoscope (cSLO), Adaptive Optics Scanning Laser Ophthalmoscope (AOSLO), Scanning Laser Polarimeter (SLP), Optical Coherence Tomography (OCT), Polarization Sensitive Optical Coherence Tomography (PS OCT), Retinal Birefringence Scanning (RBS) etc., which are the developed versions of one over other [26]. The details of each microscopy have not been discussed here.

## 5. Development of Retinal Ischemia Using Animal Stroke Models

The ophthalmic artery supplies blood supply to the retina in rodents and humans. It originates from the internal carotid artery and is proximal to the middle cerebral artery. Therefore, blocking middle cerebral artery in transient animal stroke model leads to retinal ischemia. This was first shown in the animal model of focal middle cerebral artery occlusion (MCAO) where retina ischemia was confirmed by the suppression of a waves (associated with rod photoreceptor activity) and b waves (Bipolar and muller cells) in retina [27]. Moreover, it is important to mention other sources of blood supply to ophthalmic artery which leads to retinal ischemia. As mentioned above, the origin of ophthalmic artery is internal carotid artery and internal carotid artery originally develops from the common carotid artery. Therefore, vascular obstruction of the common carotid artery in animal model of global cerebral ischemia also impairs ophthalmic blood supply, thereby developing retinal ischemia. There are reports of elimination of b wave of the electroretinogram 7 days after global cerebral ischemia in animal model [28]. Global cerebral ischemia also damages optic nerve in the long-term post occlusion [29]. Brain damage is associated stroke along with many other definite changes such as loss of retinal ganglion cells, thinning of ganglion cell complex [30] [31] [32] apart from changes in the electroretinogram in different rodent stroke models. However, there are some reports which doesn’t support the hypothesis of detectable morphological changes in the retina for animal stroke models [32] [33]. This may be due to the time duration for which ischemic event has occurred. For example, severe middle cerebral occlusion (MCAO) in rats (neuroscore > 5) only causes loss of retinal ganglion cells where less severe MCAO (neuroscore < 5) exhibits delays in the electroretinogram implicit time, which does not suggest the loss of retinal ganglion cells [34]. Retinal ischemia can also be developed by specific ligation of pterygopalatine artery which supplies blood to central ophthalmic artery [35]. However, this method can produce unspecific effects caused by physical damage caused by ligation of the pterygopalatine artery, which might not happen after stroke [28]. Although, there are other models to develop retinal ischemia such as ligation of the optic nerve, intraocular pressure elevation [36] etc. Retinal changes observed through middle cerebral artery or common carotid artery occlusion may be a better approach to induce retinal ischemia for studying pathophysiological changes after stroke because it directly blocks ophthalmic artery leaving neighboring brain structures intact. The mechanisms of retinal ischemic damage might not be the same when developed using other models. Therefore, understanding retinal changes using most relevant stroke model will be very useful in investigating therapeutics associated with stroke-induced retinal changes.

## 6. Molecular Mechanism of Parallel Retinal and Brain Changes after Stroke

Middle cerebral artery occlusion (MCAO) model is the most common method for developing ischemic stroke. The ophthalmic artery is occluded during the occlusion of middle cerebral artery causing retinal ischemia. In general, retinal ischemia in rat MCAO model causes upregulation of stress-induced proteins such as hypoxia-inducible factor alpha (HIF-*α*) and heat shock protein 70 (Hsp70), c-Fos expression [32] etc. in the retina of the affected eye. Moreover, similar change in stress induced factors such as HIF-*α* [37], Hsp70 [38] occurs in the interneurons of the hippocampus and cortex in rat model of MCAO. Based on the similar pattern of stress-induced changes in the eye and brain after stroke, eye can be used for assessing stroke induced changes. Moreover, there are also eye-specific changes such as suppression of a-(photoreceptor activity) and b-waves (depolarizing bipolar waves and potassium currents of muller cells) after MCAO model [27]. The blood-eye barrier is severely compromised in rat model of MCAO [7], which is similar to severely compromised blood brain barrier in the same animal model [39]. Moreover, there is a significant increase in the inflammatory molecules (IL-1, TNF, CCL2, MPO etc.) in the eye after rat MCAO model [7], which also occurs in the cortex in the same model. As described above in previous paragraphs, there are other models of developing retinal ischemia such as specific ligation of central ophthalmic artery or vein [40]. In these models, there was 60 % loss of retinal ganglion cells, complete loss of optokinetic response, upregulation of p-STAT and p-ERK1/2 in retinal ganglion cells and muller glia respectively [40]. Global cerebral ischemia is another simple model to mimic cardiac arrest which ultimately creates global cerebral stroke. The two-vessel occlusion for developing global cerebra ischemia also causes atrophy of the optic nerve [29] and significant reduction in the b-waves of the electroretinogram [28]. These electroretinogram changes were associated with the upregulation of glial fibrillary acidic protein (GFAP) in the muller cells [41]. As discussed above, all these changes are observed both in the retina and brain. But the question remains whether early retinal changes can be used as a biomarker for ischemic stroke in human patients? Some evidences suggest that retinal arteriovenous nicking (AVN) and retinal focal arteriolar narrowing (FAN) can predict recurrent cerebrovascular events in ischemic stroke patients [11] [42]. Thus, understanding microvascular retinal changes will give insight into novel vascular risk factor for stroke. Moreover, these retinal molecular changes in different stroke models might be worth looking as therapeutic targets for attenuating retinal damage after stroke as well because they follow the similar pattern of regulation in the eye and brain.

## 7. Therapeutics for Ischemia-Induced Retinal Damage

As discussed above, retinal damage occurs after brain stroke. This is further exacerbated by some diseases such as diabetes [43]. Diabetes retinopathy is one the leading cause of blindness across the world. Aldol reductase is known to be involved in diabetes retinopathy. Inhibition of aldol reductase reduces retinal injury in diabetic mice [24]. A hypoxic-induced chemical called semaphoring 3A prevents vascular abnormality accompanied with diabetes retinopathy. Some flavonoid drugs such as Daflon, piribedil (a D-2 agonist) reduces hydroxyl free radicals and considered neuroprotective to retinal tissues [44]. A thalidomide reduces infarct size after middle cerebral artery occlusion in mice which also preserves some of vision related circuits [45]. NR3A, a subunit of NMDA-type glutamate receptors have neuroprotective effect in cortical neurons by decreasing hyperactivation of NMDARs, thereby preventing excitotoxicity-mediated cell death [46]. The neuroprotective effect is also seen in adult mouse retina as well [46]. Moreover, retinal injury is also reduced by NF-*κ*B inhibition, a protein involved in inflammation [47]. Some innate immune system molecule such as TLR-4 deprivation reduces parenchymal stress in the retinal ganglion cells [48]. Moreover, brain-expressed X-lined (Bex) proteins (highly expressed in retinal ganglion cells) accumulates in the cytosol after optic nerve stroke [49]. The long- and short-term effects of the optic nerve stroke on the retina are observed by retinal Bex immunoreactivity [49]. Nonarteritic anterior ischemic optic neuropathy (NAION) is a type of white matter stroke resulting from optic nerve head ischemia and causes similar retinal ganglion cells apoptosis [50] as seen in other retinal ischemic models [51]. NAION leads to microglia activation and recruitment of macrophages to the infarct region [52] which also occurs in other ischemic stroke models. Thus, therapies targeting microglia and macrophages can lead to reduced RGCs apoptosis in NAION [52], which can be applied to other stroke models as well [53]. In summary, therapies which attenuate retinal damage might lead to improvements in cardiovascular and stroke treatment. This might be true otherwise as well. For example, erythropoietin has a neuroprotective effect on stroke-induced brain damage similar to the effect it has on retinal ganglion cells protection after axotomy [54]. Tacrolimus (immunosuppressant) [55] GM1 (ganglioside) [56] abrogates the ischemic-induced retinal damage, which might be useful in attenuating stroke-induced brain damage.

## 8. Conclusion

**Figure 1.**
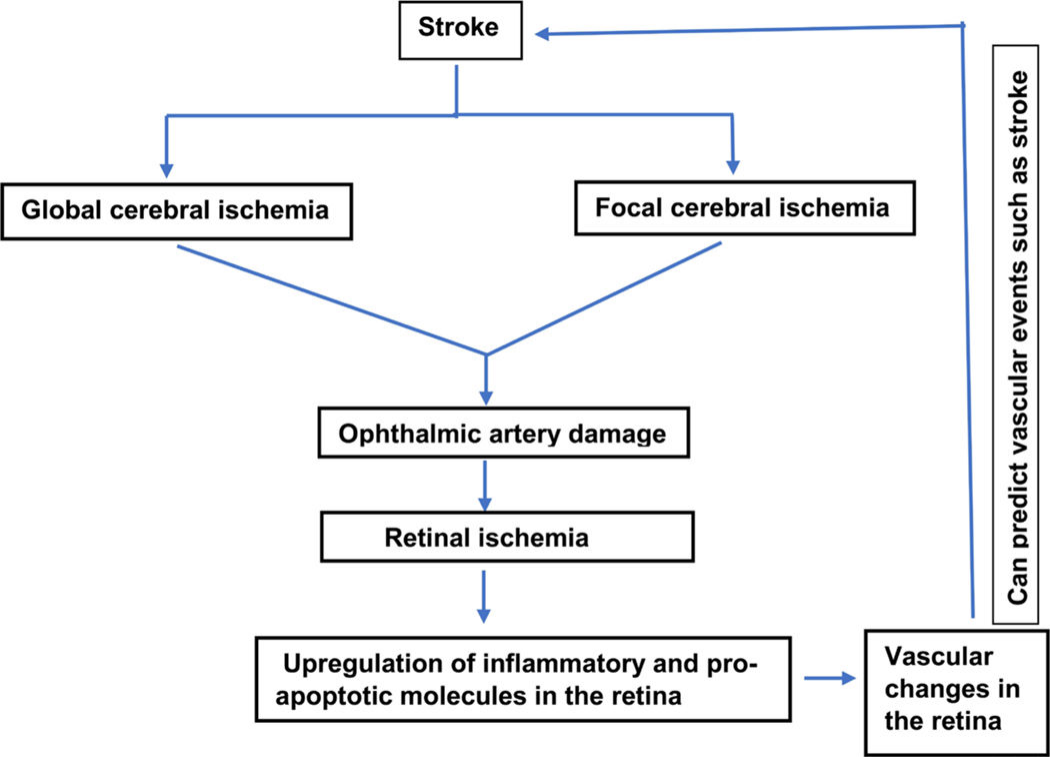
Summary of possible relationship between eye and stroke.

Stroke is a cerebrovascular accident caused by interruption of the blood supply to the brain. It is a life-threatening disease and is one of the leading causes of death and disability across the world. The functions of various organs including eye are compromised differently depending upon the affected parts of the brain after stroke and its severity. There is an urgent need to understand other similar organs which can predict the occurrence of stroke. Eye is one such organ, which is easily accessible, shares similar vasculature, anatomy and physiology and can be used to predict the occurrence of stroke. Various studies have suggested that stroke affects eye resulting in loss of vision. Having similar physiology between brain and eye, the therapeutics effective in stroke might also be useful in reducing retinal damage and vice versa. Therefore, retinal damage after stroke in clinics should not be neglected. In fact, eye should be considered as means to observe pathophysiological changes and predict stroke. A flow chart diagram has been shown for depicting the possible relationship between eye and brain pathology (Figure 1). There should be more investigations targeted at reducing retinal damage after stroke. Moreover, the molecular understandings of retinal damage after stroke and the therapeutic targets derived from it can be easily applied to ameliorate other stroke-induced brain damage.

## Figures and Tables

**Table 1.** Flow chart for the literature review.

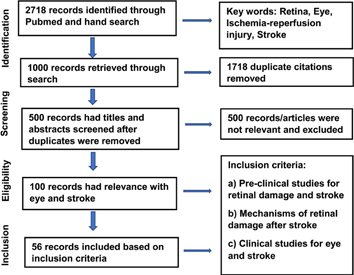

**Table 2.** Major literatures and key findings relevant for eye and stroke research

SL.NO	Year	Reference	Major Findings
1	2009	[5]	Sparser and more tortuous microvasculature network in the retina of ischemic stroke patients
2	2016	[7]	Increased inflammatory response in the retina after rodent MCAO stroke model
3	2009	[12]	Inclusion of retinal venular caliber in predicting stroke risks in human patients
4	2002	[31]	Mouse model of global cerebral ischemia causes retinal and optic nerve degeneration
5	1997	[56]	Ganglioside GM1 reduces retinal damage in mouse model of ischemic stroke.
6	2009	[47]	Astroglial NF-kappa B is detrimental to retinal neurons after rodent model of ischemic stroke
7	1995	[43]	Diabetic retinopathy is a risk factor for nonembolic stroke
8	2011	[42]	Retinal changes can predict subsequent stroke events in human patients
9	2014	[34]	More severe stroke increases the chance of retinal damage in rodent stroke model
10	2016	[21]	Age-related macular degeneration is a risk factor for stroke
